# Prediction of Age Older than 18 Years in Sub-adults by MRI Segmentation of 1st and 2nd Molars

**DOI:** 10.1007/s00414-023-03055-5

**Published:** 2023-07-04

**Authors:** Mai Britt Bjørk, Sigrid Ingeborg Kvaal, Øyvind Bleka, Tomas Sakinis, Frode Alexander Tuvnes, Mari-Ann Haugland, Heidi Beate Eggesbø, Peter Mæhre Lauritzen

**Affiliations:** 1 Institute of Clinical Dentistry, Faculty of Dentistry, University of Oslo, Postboks 1109, Blindern, N-00317 Oslo, Norway; 2 Department of Forensic Sciences, Oslo University Hospital, Postboks 4950 Nydalen, OUS, Rikshospitalet, 0424 Oslo, Norway; 3 Division of Radiology and Nuclear Medicine, Oslo University Hospital, Postboks 4950 Nydalen, OUS, Ullevål, 0424 Oslo, Norway; 4 Institute of Clinical Medicine, Faculty of medicine, University of Oslo, Postboks 4950 Nydalen, OUS, 0424 Oslo, Norway; 5 Faculty of Health Sciences, Department of Life Sciences and Health. Oslo Metropolitan University, Postboks 4, St. Olavs plass, 0130 Oslo, Norway

**Keywords:** Age estimation, Sub-adults, 1st and 2nd molars, Magnetic resonance imaging, Segmentation

## Abstract

**Purpose:**

To investigate prediction of age older than 18 years in sub-adults using tooth tissue volumes from MRI segmentation of the entire 1st and 2nd molars, and to establish a model for combining information from two different molars.

**Materials and methods:**

We acquired T2 weighted MRIs of 99 volunteers with a 1.5-T scanner. Segmentation was performed using SliceOmatic (Tomovision©). Linear regression was used to analyse the association between mathematical transformation outcomes of tissue volumes, age, and sex. Performance of different outcomes and tooth combinations were assessed based on the *p*-value of the age variable, common, or separate for each sex, depending on the selected model. The predictive probability of being older than 18 years was obtained by a Bayesian approach using information from the 1st and 2nd molars both separately and combined.

**Results:**

1st molars from 87 participants, and 2nd molars from 93 participants were included. The age range was 14-24 years with a median age of 18 years. The transformation outcome (high signal soft tissue + low signal soft tissue)/total had the strongest statistical association with age for the lower right 1st (*p*= 7.1*10^-4^ for males) and 2nd molar (*p*=9.44×10^-7^ for males and *p*=7.4×10^-10^ for females). Combining the lower right 1st and 2nd molar in males did not increase the prediction performance compared to using the best tooth alone.

**Conclusion:**

MRI segmentation of the lower right 1st and 2nd molar might prove useful in the prediction of age older than 18 years in sub-adults. We provided a statistical framework to combine the information from two molars.

**Supplementary Information:**

The online version contains supplementary material available at 10.1007/s00414-023-03055-5.

## Introduction

The rights of a child, as defined by the UN convention, applies to every human below the age of 18 years unless under the law applicable to the child, majority is attained earlier [1]. Although the age of 18 years is set, cultural traditions and social pressure may overrule legislation. Globally, girls under the age of 18 years are married off every day and may be exposed to violence and high-risk pregnancies. Refugees and asylum seekers may lack evidence of age due to incomplete birth registration, wars or poverty. In youth sports, cheating on age in order to gain advantage exists at all levels. Detection of age fraud is important to maintain the principal of fair sport and to protect the health of the competitors. Hence, age assessment is important for several purposes.

Imaging of skeletal and dental development are most commonly used in age estimation, but without a diagnostic indication it is preferable to avoid radiation of children and sub-adults [2–4]. Endocrine status, use of anabolic steroids, malnutrition, mechanical stress and injuries may influence the skeletal maturity [3]. In contrary, teeth are highly resistant to environmental and physical impact [5, 6].

The distribution of dental tissues changes throughout life. The pulp cavity decreases as an unmineralized layer of dentine matrix at the pulp surface, known as predentine, is deposited continuously, but unevenly, on the dentine inner walls. [7]. This complex process may be assessed better using tissue volumes of the entire tooth rather than linear measurements [8].

A method for dental age prediction may be more accurate when using multiple and/or different types of teeth [9]. However, statistical methods cannot completely remove the innate uncertainty associated with individual biological variation [4, 10, 11]. Agenesis, malposition, and malformed 1st and 2nd molars are rare. Hence, these teeth may be complementary or an alternative to age estimation using the recommended 3rd molars [12].

To our knowledge, no studies have been performed with in vivo tissue volume measurements from MRI of the entire 1st and 2nd molars.

Our aim was to investigate prediction of age older than 18 years in sub-adults using whole tooth tissue volumes from MRI segmentation of all the 1st and 2nd molars. We also wanted to establish a model for combining the information from two different molars.

## Material and method

Our study was approved by *the Data Protection Officer* (PVO) at Oslo University Hospital (19/10480), and the procedures were in accordance with the Declaration of Helsinki. Informed consent was signed by all participants, or their legal guardians if they were younger than 17-years-old.

This study was performed with the same participants and method as in a previous study evaluating the 3rd molars [13].

### Inclusion and Exclusion Criteria

Inclusion criteria were ages from 14 to 24 years and no contraindications to MRI acquisition according to the check list from The Norwegian Directorate of Health 2017.

Exclusion criteria for the individual molars were caries, dental fillings, erosion, excessive abrasion, and incorrect use of the dental cotton rolls and disturbing artefacts from movement or metal retainers.

### MRI Acquisition

All MRI examinations were performed using a 1.5 T scanner (Avantofit, Siemens, Erlangen, Germany) using a bilateral surface coil (Head Neck 20 and Flex Small 4 used in combination).

Our acquisition had a scan time of 5 min and 4 s and yielded 0.37-mm iso-voxels, in which a volume of 1 ml (roughly equivalent to one tooth) consists of 20.000 voxels.

Scan parameters are displayed in Table 1.

**Table 1:** MRI sequence and acquisition parameters in the MR protocol

Sequence	Time	Voxel size Reconstructed voxel size (mm)	Resolution	FOV (mm)	Number of slices	TR (ms)	TE/effective TE ms	Averages	Slab number	Flip angle
3D TSE	05:04	0.74 × 0.74 × 0.74/0.37 × 0.37 × 0.37	256 × 256	190	192	1400	182/80	1.4	1	T2 var

*TSE* turbo spin echo, *FOV* field of view, *TR* repetition time, *TE* echo time

Two cotton rolls size 2, filled with 2 ml of water, were placed bilaterally between the molars in order to displace air for better delineation of the teeth, and to stabilise the bite.

### Segmentation

The MRI examinations were separated into upper (maxillary) and lower (mandibular) teeth. Semi-automated segmentation, i.e., manually using T2 signal intensity thresholds, of the 1st and 2nd molars: 16 and 17 (upper right), 26 and 27 (upper left), 36 and 37 (lower left), and 46 and 47 (lower right), was performed on axial images in SliceOmatic (Tomovision ©, Canada). The tissue volumes were calculated in ml (cm3).

**Fig. 1:**
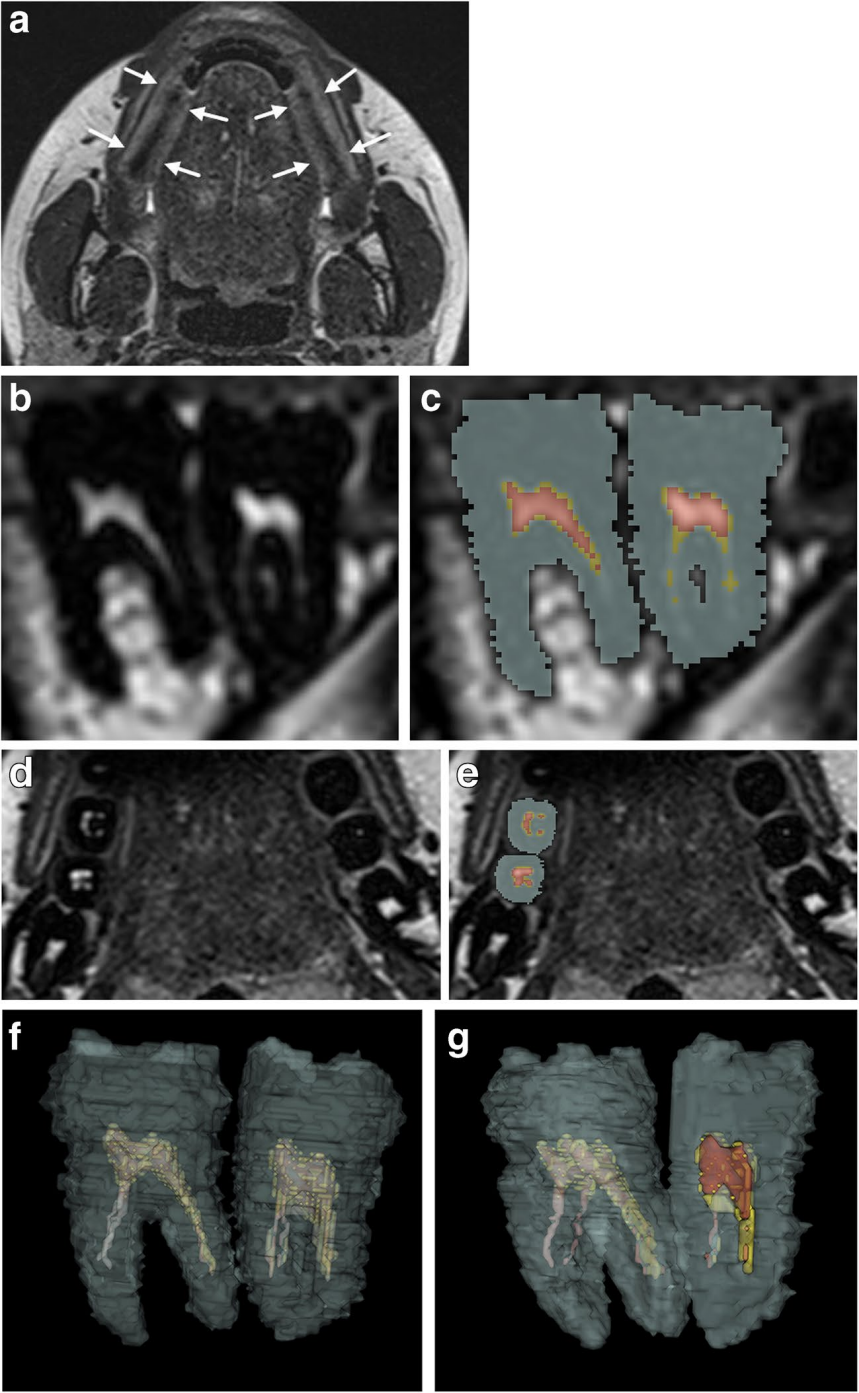
Axial MRI shows correct bilateral placement of dental cotton rolls soaked in water (arrows) between the molars (**a**). The cotton rolls delineated the upper and lower molars and stabilized the bite. Axial MRI through the upper jaw shows unsegmented (**b**) and segmented (**c**) tooth 26 and 27. Grey for hard tooth tissue, yellow for low signal soft tissue and red for high signal soft tissue. Lower and upper thresholds were set at 0 and 63 for hard tooth tissue, 64 and 100 for low signal soft tissue and ≥101 for high signal soft tissue. Sagittal MRI through the upper jaw shows unsegmented (**d**) and segmented (**e**) tooth 26 and 27. Grey for hard tooth tissue, yellow for low signal soft tissue and red for high signal soft tissue.3D of the entire tooth 46 and 47 (**f**) and 3D rendering of the segmentation (**g**), with a wedge of approximately one half removed to visualize the high signal soft tissue. Grey for hard tooth tissue, yellow for low signal soft tissue and red for high signal soft tissue

Dentine, enamel, and cementum could not be differentiated based on T2 signal intensity in our MRI sequence. These tissues were collectively segmented as hard tooth tissue, as shown in Fig. 1 b–g. Soft tissues were differentiated based on T2 signal intensity as high signal soft tissue and low signal soft tissue. Based on previous experience with ground sections, we believe that high signal soft tissue and low signal soft tissue correspond to pulp and predentine, respectively.

Lower and upper T2 signal intensity thresholds were set at 0 and 63 for hard tooth tissue, 64 and 100 for low signal soft tissue, and ≥101 for high signal soft tissue.

In order to agree on the teeth delineation and separation from surrounding tissues, a ground truth segmentation was established for the first five participants by two experienced forensic dentists and an experienced head and neck radiologist in consensus. The remaining segmentations were performed by one of the experienced forensic dentists according to the method established in consensus. The apical end of a root was defined as the point where hard tooth tissue could be identified on at least two sides, and segmentation was not performed beyond this point.

### Statistical Analyses

The association between explanatory variables (age and sex) and response variables, given as tooth tissue volumes, were analyzed with linear regression models. In addition to the three tooth tissue volumes, we explored four different transformations of the tooth tissues resulting in 10 outcomes in order to find the best response variable, as shown in Table 2. We used the natural logarithm of response variables to achieve linearity and simplify the statistical analysis.

**Table 2:** Four transformations (1–4) of the tooth tissue volumes resulting in 10 outcomes (1, and 2–4 a-c)

1) Total: high signal soft tissue + low signal soft tissue + hard tooth tissue		
2) x/(total-x)	3) x/total	4) (x + y)/Ttotal
a) High signal soft tissue/(total – high signal soft tissue)	a) High signal soft tissue/total	a) (High signal soft tissue + low signal soft tissue)/total
b) Low signal soft tissue/(total – low signal soft tissue)	b) Low signal soft tissue/total	b) (High signal soft tissue + hard tooth tissue)/total
c) Hard tooth tissue/(total – hard tooth tissue)	c) Hard tooth tissue/total	c) (Low signal soft tissue + hard tooth tissue)/total

x and y are either pulp, predentine, or dentine as shown in outcomes a-c for the transformations 2-4

Correlations between the transformation outcomes were assessed by Pearson correlation and transformations were defined as overlapping if *R* ≥ 0.999

Outcomes 2a and 2b overlapped with 3a and 3b, and outcome 2c overlapped with 4a. Hence, outcomes 2a–c were deemed redundant and not included in further statistical analysis

A large number of regression models were explored incorporating the transformation outcomes, different combinations of the four 1st and 2nd molars, different models of age and sex, and different weighting of model variance.

Age was always included as an exploratory variable in the regression model. The variable sex was incorporated into the model in five different ways: (i) Sex not considered (common intercept and age slope). (ii) Different intercepts for sex (but common age slope). (iii) Different age slopes for sex (but common intercept, age: sex). (iv) Different age slopes and intercept for sex (age **×** sex). (v) Separate model for the two sexes (same as in iv) but also different variance).

We explored three different weighting of the model variance: Either as constant (default = 1), age or (1/age).

Akaike Information Criterion (AIC) was used to select the model type for sex and variance weighting.

The 1st and 2nd molars were analyzed separately, and also evaluated in the following seven combinations (within each molar type): upper both sides, lower both sides, upper and lower right side, upper and lower left side, upper right and lower left, upper left, and lower right, all four teeth.

The performances of the transformation outcomes and tooth combinations were assessed by the *p*-value of the age variable, common or separate for each sex depending on the model. The model with the lowest *p*-value was selected and used for age prediction. For the combination of 1st and 2nd molars the transformation outcome with the best performance across the two molars were chosen.

We used a Bayesian approach to describe the uncertainty of an individual’s age [14, 15]. A prior uniform age distribution was defined from 14 to 23 years.

Posterior distribution of age after applying Bayes theorem for the best transformation outcome, in each selected tooth combination was used to estimate the probability of being older than 18 years, in each sex. This was first carried out for each 1st and 2nd molars separately, and then in combinations of the two molar types.

We assumed that the combined transformation outcome for the two molar types follow a bivariate normal model. This model includes a correlation parameter to consider statistical dependency between the two molars, as shown in the Appendix.

The model structure for the combined models was adopted from the best model when evaluating the molars separately. The parameters were estimated using maximum likelihood estimation. The data for both sexes was included into the same model in case if this was the optimal model: i.e., not being model type v). The analysis was conducted using R*v*4.2.1. The regression analysis was performed using the *lm* and *predict* function. An R-script was created for implementing the combination model where the parameters were estimated using maximum likelihood estimation. Another script was created to perform the Bayesian age predictions.

## Results

### Participants

**Fig. 2:**
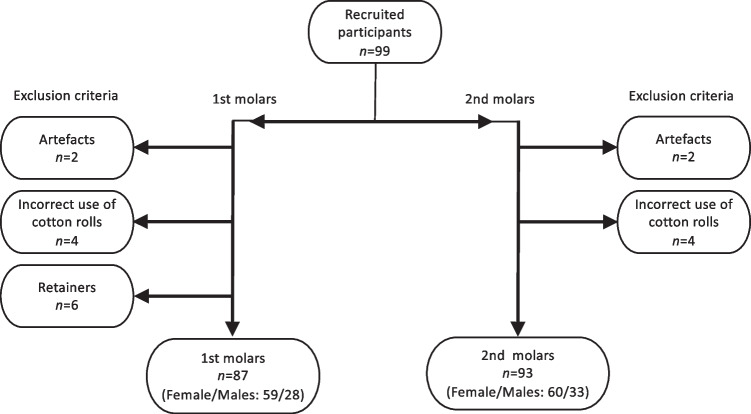
From the 99 recruited participants, six were excluded from the analysis due to movement artefacts (*n*=2) and incorrect use of cotton rolls (*n*=4). Additionally, six were excluded from the analysis of molars due to metal retainers. After exclusion, there were 87 participants (F/M: 59/28) with 1st molars and 93 participants (F/M: 60/33) with 2nd molars

**Fig. 3:**
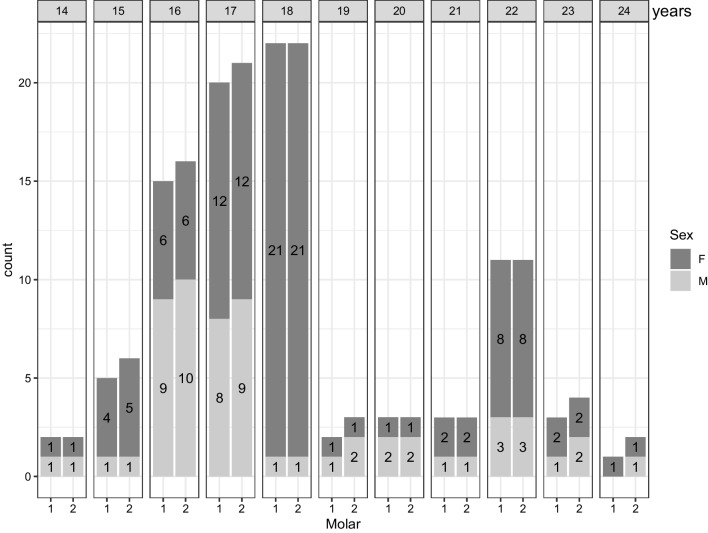
Age distribution of the 87 included participants with 1st molars and the 93 included participants with 2nd molars

After exclusion, we had 87 participants with 1st molars (F/M: 59/28) and 93 participants with 2nd molars (F/M: 60/33). Both groups with a range of 14–24 years and a median age of 18 years. Inclusion of participants is shown in Fig. 2, and age distributions are shown in Fig. 3.

Since some participants were missing tissue volumes for individual teeth due to metal retainers and incorrect placement of the cotton rolls, the number of included molars are detailed in Table 3.

**Table 3:** Distribution of included molars by sex and type of molar

Sex	Molar	Included molars (*n*)
M	16	26
M	26	26
M	36	26
M	46	25
M	17	33
M	27	30
M	37	26
M	47	29
F	16	56
F	26	58
F	36	52
F	46	50
F	17	56
F	27	58
F	37	54
F	47	56

All tissues were segmented in all included molars

### Tooth tissue volumes

The median volumes of hard tooth tissue, high signal soft tissue and low signal soft tissue are shown in Table 4.

**Table 4:** Volumes of the tooth tissues (ml)

Sex	Tissue	Median	MAD	Min	Max
1st molar					
F	Hard tooth tissue	1.1	0.13	0.70	1.4
F	HSST	0.041	0.01	0.016	0.068
F	LSS	0.021	0.0045	0.011	0.039
M	Hard tooth tissue	1.2	0.15	0.83	1.6
M	HSST	0.058	0.014	0.020	0.086
M	LSS	0.028	0.0054	0.013	0.044
2nd molar					
F	Hard tooth tissue	1.0	0.13	0.72	1.3
F	HSST	0.047	0.0099	0.020	0.080
F	LSST	0.021	0.0055	0.011	0.046
M	Hard tooth tissue	1.1	0.19	0.68	1.6
M	HSST	0.070	0.017	0.031	0.11
M	LSST	0.028	0.0079	0.011	0.053

*F* female, *M* male, *HSST* high signal soft tissue, *LSST* low signal soft tissue, *MAD* median absolute deviation, *Min* minimum, *Max* maximum

### Selected models

#### 1st molar

**Fig. 4:**
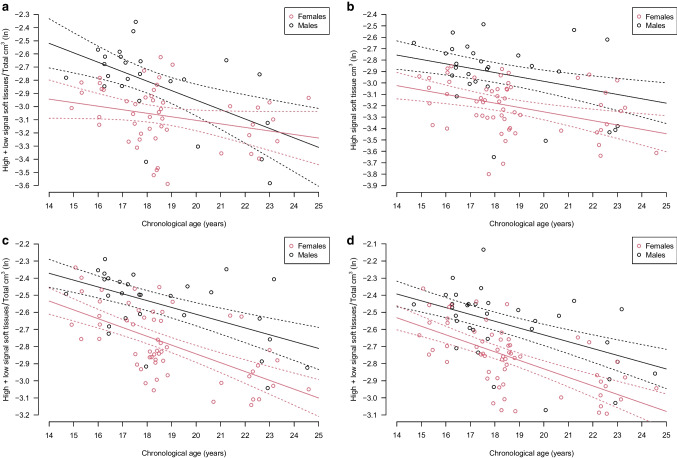
**a**–**d** The four regression models with natural logarithm of the transformation outcome (*y*-axis) with the best association with chronological age (*x*-axis). The expectations are shown as solid lines, and the 95% confidence intervals as dashed curves, red for females, black for males. Observed data are shown as circles. a The transformation outcome (high signal soft tissue + low signal soft tissue)/total, applied to tooth 46 (*p*=7.1×10^−4^) for males and *p*=0.071 for females). The sexes have different slope and intercept. **b** The transformation outcome high signal soft tissue, applied to all molars (*p*=9.4×10^-4^ for both sexes). The sexes have common slope and different intercept. **c** The transformation outcome (high signal soft tissue + low signal soft tissue)/total, applied to the best single tooth, 47 (*p*=9.4 ×10^−7^ for males and *p*=7.4×10^−10^ for females). The sexes had different slope and common intercept. **d** The transformation outcome (high signal soft tissue + low signal soft tissue)/total, applied to teeth 17 and 47 (*p*=1.9×10^−7^ for males and *p*=2.2×10^−10^ for females). The sexes had different slope and common intercept

The best performance of a single tooth was achieved by high signal soft tissue of tooth 46 in males (*p=*6.1×10^−4^). Also (high signal soft tissue + low signal soft tissue)/total for tooth 46 in males, performed well and second best (*p*=7.1×10^−4^). The regression models with different intercept and slopes (age × sex), and the variance weighting as ratio equals 1/age performed best, as shown in Fig. 4 a.

For females the best single tooth performance was achieved by low signal soft tissue for tooth 36 (*p*=1.2×10^−3^). The regression model with different slopes but common intercept (age:sex) and the variance weighting as ratio equals 1/age performed best.

The best performance overall for females was achieved by high signal soft tissue in the tooth combination of all 1st molars (*p*=9.4×10^−4^), as shown in Fig. 4b. The regression model with different intercept but common age slope (age + sex) and the variance weighting as ratio equals 1/age performed best.

#### 2nd molar

The best performance of a single tooth was achieved by (high signal soft tissue + low signal soft tissue)/total of tooth 47. This applied to both sexes (*p*=9.4**×**10^−7^ for males and *p*=7.4**×**10^−10^ for females), as shown in Fig. 4c. The regression model with different age slopes but common intercept (age:sex) and the variance weighting as ratio equals 1/age performed best.

The best performance overall for both sexes was achieved by (high signal soft tissue + low signal soft tissue)/total for the right 2nd molars (*p*=1.9×10^−7^ for males and *p*=2.2×10^−10^ for females), as shown in Fig. 4d.

The regression model with different age slopes but common intercept (age:sex) and the variance weighting as ratio equals 1/age performed best.

#### Bayesian analysis for age assessment

**Fig. 5:**
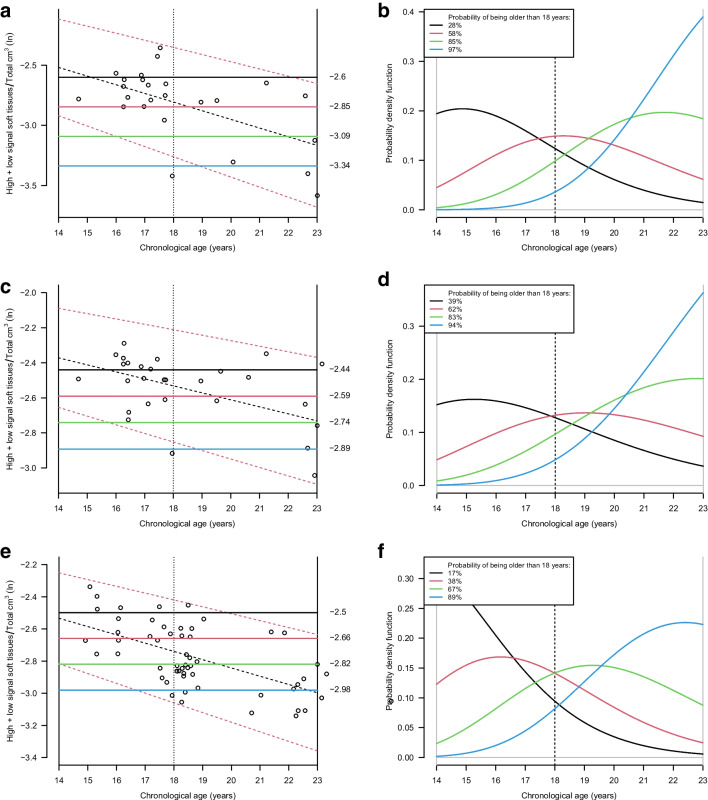
a–**f** Bayesian analysis for age assessment. Tooth 46 in males (**a**) and (**b**), tooth 47 in males (**c**) and (**d**), and tooth 47 in females (**e**) and (**f**). Between the actual minimum and maximum observations for (high signal soft tissue + low signal soft tissue)/total, four hypothetical observations were placed in uniform intervals (solid horizontal lines colour coded black, red, green and blue). For the selected model, these hypothetical observations were used as examples to illustrate the probability of an individual being older than 18 years given the observed tooth tissue volumes. The expectation of an individual being older than 18 years is shown as dashed black oblique line for males in (**a**) and (**c**), and for females in (**e**). The 95% prediction interval of volume measurements is shown as dashed red oblique lines of the natural logarithm (high signal soft tissue + low signal soft tissue)/total on the y-axis, applied to tooth 46 (**a**), and 47 (**c**) in males and tooth 47 in females (**e**) against chronological age (x-axis). The limitation of the prior age distribution (14.0–23.0 years) is shown as vertical, solid black lines. The 18-year-threshold is shown as a vertical, dashed line. The posterior age distributions for males (**b**) and (**d**) and females (**f**), after applying Bayes theorem by assuming a uniform prior. The age distribution curves are color-coded black, red, green and blue, and correspond to the hypothetical ratios in (**a**), (**c**), and (**e**). The probabilities of being older than 18 years for each hypothetical ratio are shown in the legends. The area under each curve is 100% of all probabilities (area equal to 1). The age distribution curves are limited by the prior age distribution (14.0–23.0 years), shown as vertical, solid black lines. The 18-year-threshold is shown as vertical, dashed line

The transformation outcome (high signal soft tissue + low signal soft tissue)/total for tooth 46 in males and tooth 47 for both sexes were used for illustration since this outcome would be one of the top models for dental age prediction, as shown in Figs. 5a–f.

#### Combination of 1st and 2nd molar

To illustrate how two molars can be combined we used the teeth 46 and 47 for males and the transformation outcome (high signal soft tissue + low signal soft tissue)/total. We chose this combination for illustration because it showed the best performance across the two molars.

For tooth 46 the model variant with different intercept and age slope for sex was selected, while for tooth 47 we assumed different slopes, but common intercept.

The maximum likelihood estimate of the correlation parameter was 0.62 (Supplementary Table). The Bayesian framework was applied to make age prediction. The same four hypothetical values were considered as in Fig. 5, but now they were applied for two molars combined.

**Fig. 6:**
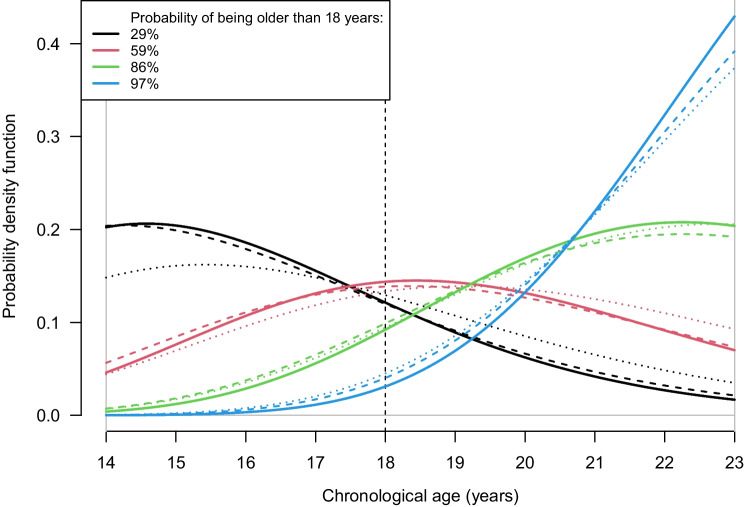
The combination of tooth 46 and 47 in males. The natural logarithm for (high signal soft tissue + low signal soft tissue)/total is shown as solid curves for the posterior age distributions, obtained by applying Bayes theorem, with a uniform prior age distribution (14.0–23.0 years). Posterior age distribution for tooth 46 is shown as dashed curves, and for tooth 47 as dotted curves. The same hypothetical observations, as shown in Fig. 5 a and c, were used as examples to illustrate the probability of an individual being older than 18 years given the observed tooth tissue volumes. The probability of being older than 18 years by combining tooth 46 and 47 is shown in the legend

The age prediction distributions and the probabilities of being older than 18 years for 1st and 2nd molar separately and combined for males are shown in Fig. 6. The probability of being older than 18 years did not change much when combining the two molars compared to each molar separately. The values for the hypothetical measurement for the highest probability did not change (97%), whereas the value for the second highest probability changed from 85 to 86%.

## Discussion

We have developed a fast and simple in vivo MRI protocol, and a method for segmentation of whole tissue volumes of all the 1st and 2nd molars.

There was a very strong association between age and tooth tissue development of the lower right 2nd molar for both sexes, and a strong association for the 1st molar in males. There is no obvious biological reason why the tissue volume of one specific 2nd molar should have a stronger association with age than others, and indeed the correlation was very strong for the remaining 2nd molars also. However, to show the potential of this approach, we based our model to predict the probability of being older than 18 years on the molar with the strongest association.

To our knowledge our study is the first to develop a statistical framework for age prediction which combine information from multiple molars taking dependency into account. We built a model for the combination of the 1st and 2nd molar, and provided an illustrative example combining the best model for each molar in males.

Previous studies that have used 1st molars with cone beam computed tomography (CBCT) and orthopantomogram (OPG) have concluded that the pulp chamber volume is a useful index for age prediction [9, 16]. The pulp/tooth ratio of 2nd molars using OPGs has been shown to be an appropriate indicator of adult age [17]. Using CBCT on the same tooth measuring the pulp chamber volume may be a relatively accurate indicator for the same purpose [8].

### MRI acquisition and segmentation

We have previously applied our customized 1.5 T MRI acquisition and tooth tissue segmentation method to 3rd molars [13]. Two other studies have managed to determine pulp cavity volume on extracted teeth with a 9.4 Tesla, but the field of view and spatial resolution had to be adjusted for each different type of tooth [18, 19].

We segmented dentine, enamel, and cementum collectively as hard tooth tissue since they all have very low T2 signal intensity and could not be differentiated on our MRI acquisition. However, we could differentiate hard tooth tissue from high signal and low signal soft tissue, and establish T2 signal intensity thresholds for tissue-segmentation of the entire 1st and 2nd molars. Even with signal intensity thresholds, segmentation still requires some anatomical knowledge. Nevertheless, we regard it as more objective than grading of root development.

The tissue segmentations were not histologically confirmed in this cohort since the image acquisition was in vivo. However, from previous experience the tissue volumes segmented with this method correspond to those on ground sections, and the strong correlation with age further support that the segmentations correspond to developing tissues.

To our knowledge no previous study has performed in vivo full volume measurements of the entire 1st and 2nd molars. Other studies using CBCT on 1st and 2nd molars have set the pulp chamber floor as the “cut off plane” and excluded the roots [9]. In our study, segmentation was performed in the axial plane; however, the high-resolution 3D acquisition with isotropic voxels allows for reconstruction with equal resolution, and thus segmentation, in any plane.

### 1st and 2nd molars and models

We found a strong association between 1st molar high signal soft tissue volume and age, which is in accordance with previous studies showing that pulp decreases with advancing age [20]. The very strong association between tooth tissue development and age obtained for the 2nd molar agrees well with a previous study reported that the maxillary 2nd molar was best suited for age estimation based on pulp chamber/cavity volume [9]. Fortunately, 2nd molars are less affected by retainers than the 1st molars and have a simpler root anatomy and are less prone to agenesis than 3rd molars [21]. The 2nd molars also have less caries and tooth wear since they erupt at an older age than the 1st molars.

Using both 1st and 2nd molars resulted in a larger material, in which the 2nd molars were most numerous due to less affection by metal retainers.

In our study, the transformation outcome (high signal soft tissue + low signal soft tissue)/total performed well for both sexes. Nevertheless, it is preferable to include sex as a parameter in age estimation models, since our data and other studies have shown varying degrees of differences between the sexes [8, 9, 22, 23].

The added value of combining 1st and 2nd molars in the same model was very small since the probability estimates barely changed compared to considering the molars alone. Further improvements might well be achieved by adding the 3rd molar or other teeth to the model. In the combined illustration example in males we found that tooth 46 provided slightly more information in the age prediction compared to tooth 47. This is somewhat unexpected, given that the association with age was stronger for tooth 47 than for tooth 46, in terms of *p*-values. This is probably due to slight differences in the number and age distribution of participants in the model for these two teeth. In the combined illustration example, there were four less samples for tooth 46 than tooth 47, and one of these was at age 24, which affects the calculated *p*-value.

### Statistical analysis

We used a Bayesian approach to avoid age mimicry [14, 15].

The age priors in this study was aligned with a previous study 3rd molar tooth tissues in order to achieve consistent and comparable results, and to facilitate future prediction models combining all three molars [13]. Hence, the lower prior was given by 3rd molar development and set at 14 years, and upper prior was chosen at 23 years, compared to 20.5 years for BioAlder [12]. Increasing the upper prior, increases the risk of falsely classifying a person as older than 18 years.

Our mathematical framework and models enabled combination of information from the 1st and 2nd molars even for individuals where single or multiple molars were excluded.

## Limitations

Since there was limited ethnic variation in our study population, the validity of the models in other ethnic groups is uncertain [24]. Excessive tooth wear, caries, and dental fillings were not present in our study population. Since MRI is prone to artefacts from different dental filling materials [19], our method might not be optimal in subjects with a low socioeconomic status [25–27]. Further, our study population was relatively small, and our results have not been validated in an independent cohort.

Combining this method with other physical traits (teeth, hand, clavicle, and DNA methylation) is recommended for legal purposes, and may reduce the uncertainty due to biological variation and increase the robustness of the method to missing teeth [28]. However, the optimal combination of dental and other biological traits in age prediction remains to be established.

## Conclusion

MRI segmentation of the lower right 2nd molar tissues might prove useful in the prediction of age older than 18 years in sub-adults. We also found that the lower right 1st molar tissue may be useful for males. Our novel application of a statistical framework for dental age prediction allows for combination of information from multiple molars with dependency taken into account.

## Supplementary information

ESM 1 (DOCX 16 kb)

ESM 2 (PDF 42.6 KB)

## Data Availability

All data was registered, including data that was deleted or changed. Anonymised data was exported for statistical calculations. After database lock, the data was saved according to current regulation.

## Abbreviations

*CBCT*: Cone beam computed tomography
*CT*: Computed tomography
*DCNN*: Deep convolutional neural network
*FOV*: Field of view
*FSE*: Fast spin echo
*HSST*: High signal soft tissue
*LSST*: Low signal soft tissue
*MRI*: Magnetic resonance imaging
*OPG*: Orthopantomogram
*TE*: Echo time
*TR*: Repetition time
*TSE*: Turbo spin echo
*T2*: Transverse relaxation time
*UN*: United Nations
*16*: Upper right 1st molar
*26*: Upper left 1st molar
*36*: Lower left 1st molar
*46*: Lower right 1st molar
*17*: Upper right 2nd molar
*27*: Upper left 2nd molar
*37*: Lower left 2nd molar
*47*: Lower right 2nd molar
