# Supplementary material for: Prediction of Age Older than 18 Years in Sub-adults by MRI Segmentation of 1st and 2nd Molars
Source: Int J Legal Med. 2023 Jul 4;137(5):1515–26. doi: 10.1007/s00414-023-03055-5 (PMC10421773; doi:10.1007/s00414-023-03055-5)
Supplement: Supplementary file 1 — (DOCX 16 kb) [file 414_2023_3055_MOESM1_ESM.docx]

# Appendix: Mathematical description of the combination model

## The likelihood function: Used for estimating parameters

We implemented a model to combine information in method 1 and method 2, in such a way that all individuals are utilized in the estimation of the parameters, even if they have missing values for one of the methods. This can be done by splitting the likelihood function into three parts:

$$L\left( \theta\right)= L_{1}\left( \theta_{1} \right)L_{2}\left( \theta_{2} \right)L_{12}\left( \theta_{12} \right)$$

$L_{1}\left( \theta_{1} \right)$ and $L_{2}\left( \theta_{2} \right)$ are the marginal likelihood expressions for method 1 and method 2, where the individuals are only recorded with measurements for either method 1 or method 2, respectively. $L_{12}\left( \theta_{12} \right)$ is the likelihood based on the bivariate normal model where individuals are recorded with both measurements. The unknown parameters consist of$\theta=(\beta_{1},\sigma_{1},\beta_{2},\sigma_{2},\rho)$: $\beta$ includes both the intercept and slope parameters (and possibly additional for each sex), $\sigma$ is the standard deviation, with index indicating the method (1 or 2) and $\rho$ is the correlation parameter. The subscript indicates which method the parameters belong to:$\theta_{1}=(\beta_{1},\sigma_{1})$, $\theta_{2}=(\beta_{2},\sigma_{2})$ and $\theta_{12}= \theta$ (all parameters used). We estimated the unknown parameters $\theta$ using maximum likelihood estimation:  $\hat{\theta}=argmax_{\theta}L\left( \theta\right).$ Further follows more details about the underlying model.

## The underlying model for observations: Used to define the likelihood function

We define $Y_{1}$ and $Y_{2}$ as stochastic variables for the volume measurements of method 1 and 2, with individual observations for individual $i$ for method $m$ given as $y_{i,m}$. By inserting the volume measurements for individual of method $m$ into the probabilistic model (defined below) we obtain the likelihood function as

$$L_{m}\left( \theta_{m} \right)=\prod_{i=1}^{n_{m}} P\left( Y_{m}=y_{i,m} \right|\theta_{m})$$

where $n_{m}$ is the number of individuals recorded only for a method $m$. The likelihood function for the joint model is given as

$$L_{12}\left( \theta_{12} \right)=\prod_{i=1}^{n_{12}} P\left( Y_{1}=y_{i,1},Y_{2}=y_{i,2} \right|\theta_{m})$$

where $n_{12}$ is the number of individuals recorded for both methods.

We further introduce the design matrix $X$ which includes explanatory information about the individuals: a factor for sex and a numerical value for age. The $i$th row of the matrices, $X_{i,1}$ and $X_{i,2}$, are the explanatory variables for individual $i$, for methods 1 and 2, respectively. From this the probabilistic models are defined as a bivariate normal distribution (Multivariate Normal with dimension 2):

$$P\left( Y_{1}=y_{i,1},Y_{2}=y_{i,2} \right|\theta_{12})= MVN_{2} \left( \left[ \begin{matrix} \begin{matrix} y_{i,1} \\ y_{i,2} \end{matrix} \end{matrix} \right]| \left[ \begin{matrix} \begin{matrix} X_{i,1}\beta_{1} \\ X_{i,2}\beta_{2} \end{matrix} \end{matrix} \right],\left[ \begin{matrix} \sigma_{1}^{2}w_{i} & {\rho\sigma_{1}\sigma}_{2}w_{i} \\ {\rho\sigma}_{1}\sigma_{2}w_{i} & \sigma_{2}^{2}w_{i} \end{matrix} \right] \right)$$

where we in our implementation assume that the variance weight is given as $w_{i}=age_{i}$ (this corresponds to the best AIC model). The marginal models are defined as

$$P\left( Y_{m}=y_{i,m} \right|\theta_{m})=N\left( y_{i,m} | X_{i,m}\beta_{m},\sigma_{m}^{2}w_{i} \right).$$

## Prediction of new individuals utilizing combined measurements

From the previous section we defined a joint model for observed measurements on two methods ($y_{1}$and$y_{2}$): Since age is an independent variable as part of the design matrix $X$ we have that, for a given sex,

$$P\left( Y_{1}=y_{1},Y_{2}=y_{2} \right|\theta_{12})=P\left( Y_{1}=y_{1},Y_{2}=y_{2} \right|\theta_{12},Age=a)$$

By applying Bayes theorem, we obtain an expression for the posterior distribution of age:

$$P\left( Age=a \right|y_{1},y_{2})= c*P\left( Y_{1}=y_{1},Y_{2}=y_{2} \right|\theta_{12}=\hat{\theta},Age=a_{i})P(Age=a)$$

where $P(Age=a)$ is the prior distribution of age for a new predicting individual, and $c$ is a normalization constant. We use the maximum likelihood estimate of the parameters as fixed values for the unknown model parameters.
