# Supplementary material for: Prediction of Age Older than 18 Years in Sub-adults by MRI Segmentation of 1st and 2nd Molars
Source: Int J Legal Med. 2023 Jul 4;137(5):1515–26. doi: 10.1007/s00414-023-03055-5 (PMC10421773; doi:10.1007/s00414-023-03055-5)
Supplement: Supplementary file 2 — (PDF 42.6 KB) [file 414_2023_3055_MOESM2_ESM.pdf]

Supplementary table The estimated parameters for the combined model versus the marginal models for the best transformation of the 46 and 47 molars. The correlation for marginal model were calculated from the residuals.

	**Combined**	**Marginal**
46: Intercept (common)	-1,71	-1,513
46:+ Intercept Female	-0,845	-1,046
46: Age slope Male	-0,061	-0,072
46: Age slope Female	-0,028	-0,027
46: Variance	0,053	0,053
47: Intercept (common)	-1,794	-1,816
47: Age slope Male	-0,041	-0,04
47: Age slope Female	-0,052	-0,051
47: Variance	0,037	0,038
Correlation (46 vs 47)	0,615	0,577
